# Evaluation of *In Vitro* Antiprotease Activity of Selected Traditional Medicinal Herbs in Dentistry and Its In Silico PASS Prediction

**DOI:** 10.1155/2022/5870443

**Published:** 2022-06-06

**Authors:** Ali A. Assiry, Shaeesta Khaleelahmed Bhavikatti, Fahad A. Althobaiti, Roshan Noor Mohamed, Mohmed Isaqali Karobari

**Affiliations:** ^1^Preventive Dental Science Department, Faculty of Dentistry, Najran University, Kingdom of Saudi Arabia, Saudi Arabia; ^2^Department of Periodontics, Universiti Sains Malaysia, Health Campus, Kubang Kerian, Kota Bharu, 16150 Kelantan, Malaysia; ^3^Center for Transdisciplinary Research (CFTR), Saveetha Dental College, Saveetha Institute of Medical and Technical Sciences, Saveetha University, Chennai, Tamil Nadu 600077, India; ^4^Pediatric Dentistry Resident, Ministry of Health, Saudi Arabia; ^5^Department of Pediatric Dentistry, Faculty of Dentistry, Taif University, PO Box 11099, Taif 21944, Saudi Arabia; ^6^Department of Restorative Dentistry & Endodontics, Faculty of Dentistry, University of Puthisastra, Phnom Penh 12211, Cambodia

## Abstract

**Background:**

Dental/oral diseases are one of the significant public health problems globally. Herbal medicines for managing oral diseases are considered an effective alternative to synthetic compounds due to their lower side effect. *Azadirachta indica, Terminalia chebula, Camellia sinensis,* and *Piper nigrum* are used to control and prevent oral inflammations in dentistry. In this study, we have evaluated the protease inhibition activity of these plant extracts, and further, the binding mode of the active ingredient of these plants with trypsin was studied using molecular docking.

**Methods:**

In this study, protease inhibition activity was carried out using aqueous extracts of the plant parts such as *Azadirachta indica* (neem) twig, *Terminalia chebula* (Haritaki) fruit, *Camellia sinensis* (green tea) powder, and *Piper nigrum* (kali miri) seed. Next, to explore the binding mode of active ingredients azadirachtin, chebuligenic acid, catechin, and piperine with trypsin, we employed a molecular docking study using AutoDock4.2.

**Results:**

The results revealed that the *Azadirachta indica plant* extract showed an IC50 value of 96.19 *μ*g mL^−1^, *Camellia sinensis* IC50 value of 188.50 *μ*g mL^−1^, *Piper nigrum* IC50 value of 371.20 *μ*g mL^−1^, and *Terminalia chebula* IC50 value of 639.48 *μ*g mL^−1^, when compared with standard drug diclofenac sodium, had IC50 value 93.00 *μ*g mL^−1^. Further, the docking result reveals that all the main active ingredients of these plants have significant binding affinity and prefer the same binding pocket of trypsin.

**Conclusion:**

Hence, our results show the importance of traditional plants *Azadirachta indica*, *Terminalia chebula*, *green tea*, and *Piper nigrum* to control oral disease conditions. As they show significant protease inhibition activity, hence, the active ingredient could act as a potential anti-inflammatory agent and further help to prevent or control oral disease conditions such as gingivitis and periodontitis.

## 1. Introduction

Human history has shown a broad spectrum of therapeutic development, and the past shows that humans have always been fond of nature. Traditional medicine (TM) is considered the oldest form of healthcare [1]. According to the World Health Organization, 80% of the world's people depended on TM (herbal) for their primary healthcare needs as these plant extracts were readily accessible, affordable, and culturally appropriate [2]. Traditional medicine was initially used to treat medical conditions; however, there was an emerging trend of traditional medicine used in dentistry to relieve tooth pain, endodontic inflammation, periodontal inflammation, and oral mucosal disease [3]. Characteristically, two common diseases are affecting the oral tissues and the health of the supporting structures of a tooth. The inflammation of the soft tissues, epithelium, connective tissue, or the inflammatory processes extend to the periapical and involve the supporting tissues, including the alveolar bone [4]. However, the pathological consequences are triggered by the accumulation of bacteria at the tooth surface, leading to a host response generating inflammatory cell infiltration [5]. Indigenous people used natural toothbrushes made from healing plants to brush their teeth [3]. Another study conducted in the United States showed that those that could not afford to visit dentists might use alternative sources of care for toothache pain relief [6]. There are many natural ways and herbs to treat dental inflammatory conditions and infections, some even help prevent them. Twig is used for toothbrushing; it contains volatile oils which stimulate blood circulation and tannins that cleanse gums.

The upsurge in the use of herbal medicines has accelerated the interest in the effects of plant extracts in the management of oral diseases. The potential role of plant extracts, essential oils, and natural compounds as antibiofilm agents in dentistry, and the quest for natural anti-inflammatory agents with minimal adverse effects have paved the way toward the development of efficacious herbal medicines. Different natural common herb components such as tea tree oil, aloe vera, vanillin, curcumin, and chamomile, have been recently been explored as anti-inflammatory molecules for dental treatments [7].

Commonly used are neem twigs (*Azadirachta indica*). *Azadirachta indica* mouth wash is reported to inhibit the growth of *S. mutans* and carious lesions [8]. Triphala, as it implicates, is an equal combination of three important ayurvedic herbs, Amalaka (*Emblica officinalis*), haritaki (*Terminalia chebula*), and bibheetaka (*Terminalia bellirica*). *Terminalia chebula* herbal extract effectively inhibits biofilm formation, and its better antioxidant activity, which is exhibited by this extract, could protect the gingival cells effectively from free radicals than the commercial kinds of toothpaste. Thus, *Terminalia chebula* could be used as an effective antiplaque agent [9]. Existing literature emphasizes the beneficial properties of green tea, including its effectiveness as an antimicrobial to pathogenic bacteria and oral fungi, which can be used in clinical practice to treat and prevent oral cavity disorders [10]. *Piper nigrum* (black pepper) is known as one of the world's most relevant spices due to its high content of piperine [11]. Thus, four important medicinal herbal plants with known significant benefits were selected for the study.

Various protease enzymes are involved in many essential intra- and extracellular physiological processes. A plethora of evidence is available that indicates proteases can regulate their target cells by activating and breaking proteases-activated receptors (PARs). Significant roles for PARs in inflammation have also been proposed. Recent reports have revealed that protease inhibitors may have anti-inflammatory roles apart from mere suppressive effects on protease actions during inflammation [12]. Though several anti-inflammatory drugs are available, including nonsteroidal anti-inflammatory drugs (NSAIDS), they are associated with adverse effects, with unwanted effects on the gastrointestinal tract, the kidney, and the cardiovascular system [13].

A paucity of the literature suggests the protease inhibitory effects of the four essential herbs that could be used effectively as anti-inflammatory agents, especially in managing dental inflammatory conditions. With this viewpoint, the purpose of the present investigation was to evaluate the anti-inflammatory potential of the selected medicinal plants by protease inhibition assay, which was further confirmed by molecular docking studies.

Thus, the present investigation scrutinizes the active ingredients such as azadirachtin, chebuligenic acid, catechin, and piperine from the *Azadirachta indica*, *Terminalia chebula, green tea*, and *Piper nigrum,* respectively, inhibit the protease, an enzyme that is responsible for inflammation. We hypothesized that these traditional plants have potential bioactive which could act as anti-inflammatory agents; hence, the present study focuses on the analysis of active ingredients which could be further developedas anti-inflammatory drugs for oral diseases.

## 2. Materials and Methods

In the present study, we have collected traditional plants used in the dentistries for oral disease conditions. The plant materials were further processed to extract and the biological activity was evaluated using the protease inhibition assay. Further, the binding mode of active ingredients was evaluated using the molecular docking study. The details of the study are given below.

### 2.1. Collection of Plant Material and Processing

Plant parts of *Azadirachta indica* twig, *Terminalia chebula* fruit, *Piper nigrum* seed, and *Camellia sinensis* were collected. The plants were identified and authenticated by an approved botanist, and a voucher specimen of each plant was deposited in the department. Plant materials were washed with tap water and dried in an oven at 45°C for seven days. The material was ground; the fine powder was made and stored in an air-tight container until use.

### 2.2. Reagents Used

Trypsin, Tris-HCl, perchloric acid, and casein were obtained from Sigma Aldrich, St. Louis, MO, USA. All other chemicals used were of analytical grade.

### 2.3. Preparation of Plant Extracts

16 gm of twig powder of neem, fruit powder of haritaki, seed powder of kali miri, and green tea powder at 40°C for 4 h then at room temperature for 24 h was filtered and smoothly evaporated under vacuum. The extracts were stored at 40°C until further use [14].

### 2.4. Protease Inhibition Assay

For the evaluation, aqueous extracts of a twig of *Azadirachta indica* twig, *Terminalia chebula* fruit, *Piper nigrum* seed, and *Camellia sinensis* in vitro protease inhibition assay was carried out. In proteinase inhibition assay, diclofenac sodium was used as a standard. For the reaction mixture of 2.0 mL consisting of 250 *μ*L of trypsin & 1.0 mL 25 mM Tris-HCl buffer (pH 7.4) and 1.0 mL of an aqueous solution of each extract (100-1000 *μ*g/mL) were added accordingly. The mixture was incubated at 37°C for 5 minutes. 1.0 mL of 0.8% (*w*/*v*) casein was added to each extract. The mixture was incubated for an additional 20 minutes. 2.0 mL of 70% (*v*/*v*) perchloric acid was added to each extract to terminate the reaction. The cloudy suspension was centrifuged, and the absorbance of the supernatant (protein hydrolyzed) was determined at 280 nm against the buffer as a blank.

The experiment was performed in triplicate. The percentage inhibition of proteinase inhibitory activity was calculated according to the formula: Percentage proteinase inhibitory action = (absorbance blank − absorbance sample) ×100/absorbance blank. The IC50 (concentration providing 50% of proteinase inhibitory action) values were calculated using the dose inhibition curve in a linear range by plotting the sample concentration versus the corresponding proteinase inhibitory action [15]. The trypsin enzyme was used for the inhibition assay in the present study. Hence, molecular docking was performed to investigate the binding mode and interactions of active ingredients such as azadirachtin, chebuligenic acid, catechin, and piperine with trypsin enzyme. The detailed docking protocol is discussed below.

### 2.5. Molecular Docking

Molecular docking was performed to investigate the binding mode and interaction of trypsin protein with azadirachtin, chebulagic acid, catechin, and piperine, which are the main ingredient of the plant *Azadirachta indica* [16, 17], *Terminalia chebula* [18], green tea [19], and *Piper nigam* Linn [20]. We have shown that the plant, as mentioned above, extract has proteinase inhibition activity. We have used the trypsin enzyme for proteinase inhibition assay; therefore, the binding mode of azadirachtin, chebulagic acid, catechin, and piperine were explored by using the docking study. For molecular docking, we used AutoDock4.2 [21], which is widely used to investigate the putative binding mode of the ligand with receptor target structure [22, 23].

The crystal structure of trypsin protein (source code: 1TRN.pdb) was taken from the protein database (http://www.rcsb.org/). For docking, three-dimensional atomic coordinates of azadirachtin, chebulagic acid, catechin, and piperine were built using the Avogadro software [24]. The putative binding mode of the drug mentioned above was investigated using the blind and local docking approaches similar to earlier studies [23]. Here, a blind and local docking approach (http://autodock.scripps.edu) was used to explore the putative binding site of the abovementioned compound with trypsin. The Lamarckian genetic algorithm was utilized with the default parameters, similar to earlier studies [23]. The output conformations of drug compounds were further clustered using an all-atom RMSD with a cut-off of 4 Å, similar. These resulted output conformations were then compared based on the cluster size, intermolecular energy, van der Waals energy, and binding energy similar to previous studies [22, 25]. The lowest binding energy conformation was further analyzed for hydrogen bonding, van der Waals, and electrostatic interactions using the Discovery Studio visualize [26] and PyMo [27] software.

## 3. Results

### 3.1. Extraction and Yield of the Medicinal Plant

**Figure 1:**
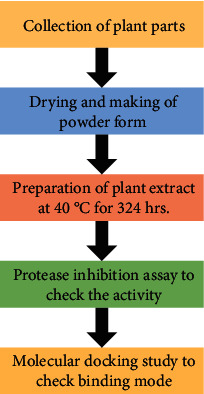
Flow chart of the study design to evaluate the anti-inflammatory activity of the plant extract.

The four different aqueous extracts were collected as brownish solid (Table 1), and the yield of the *Azadirachta Indica* twig extracts was 3.22 g (yield 20.12%), *Terminalia chebula* (haritaki) fruit extract was 2.28 (yield 14.25), *Piper nigrum* (kali miri) seed extract was 3.10 (yield 19.37%), and *Camellia Sinensis* powder extract is 3.96 (24.75%). The yields of the selected extracts are shown in Table 1. Hence, these extracts were further used for the protease inhibition assay and discussed below. The flow chart of the study to evaluate the anti-inflammatory activity is shown in Figure 1 for more details.

### 3.2. Protease Inhibition Assay

The aqueous extracts of different plant parts at different concentrations (dose levels) provided significant inhibition of proteinase assay. The maximum (%) inhibition was observed in *Azadirachta indica* Linn, *Camellia sinensis* Linn, and *Piper nigrum* Linn extracts. All these plant extracts possessed a significant IC50 value of *Azadirachta indica* Linn (96.19 *μ*g mL^−1^), *Camellia sinensis* Linn (188.50 *μ*g mL^−1^), and *Piper nigrum* Linn (371.20 *μ*g mL^−1^) activity comparable to that of diclofenac sodium (93.00 *μ*g mL^−1^). *Terminalia chebula* Retz fruit extract showed moderate activity against protease inhibition assay (Table 2).

### 3.3. Molecular Docking

**Figure 2:**
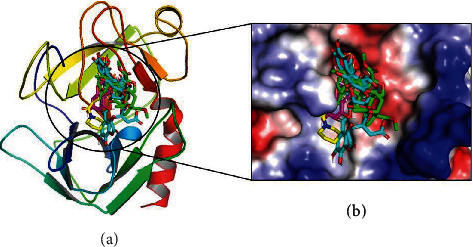
Docking of trypsin with azadirachtin, chebulagic acid, catechin, and piperine. (a) The binding mode of azadirachtin (green), chebulagic acid (cyan), catechin (magenta), and piperine (yellow) with trypsin. (b) The solvent-accessible surface area of the trypsin binding pocket with the docked structure of azadirachtin, chebulagic acid, catechin, and piperine. Here, all the compounds prefer the same binding site of the trypsin receptor.

Molecular docking was performed to investigate the putative binding mode of azadirachtin, chebulagic acid, catechin, and piperine compound with trypsin receptor using AutoDock4.2 [21]. Here, docking analysis reveals that these compounds prefer the same binding site of the trypsin as shown in Figure 2. The least binding energy docked conformation of azadirachtin, chebulagic acid, catechin, and piperine with trypsin receptor was -5.93 kcal mol^−1^, -6.14 kcal mol^−1^, -7.57 kcal mol^−1^, and -8.36 kcal mol^−1^, respectively (Figure 2(b) and Table 3). Furthermore, interaction analysis was performed to explore the residues involved in binding the compounds as mentioned above.

**Figure 3:**
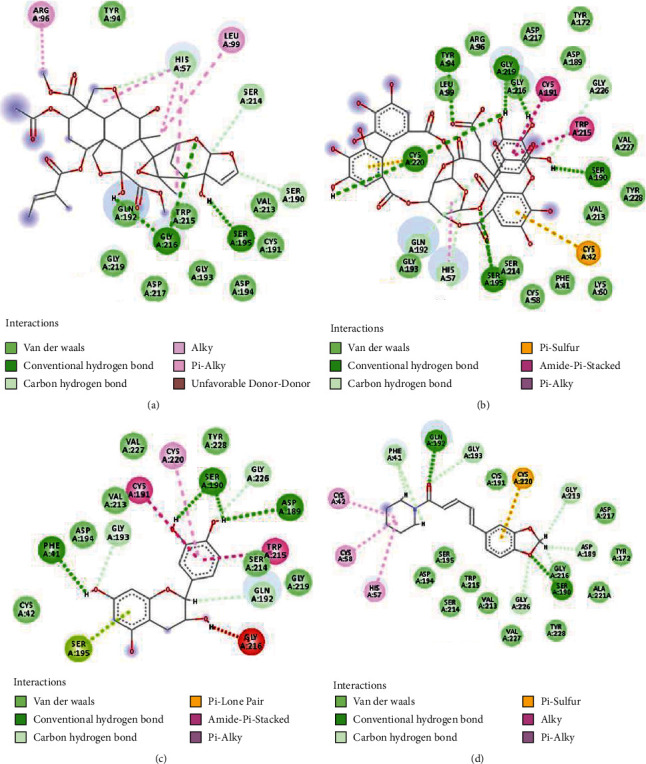
The 2D interactions of trypsin residues with azadirachtin, chebulagic acid, catechin, and piperine. (a) The interactions of trypsin residues with azadirachtin. (b) The interactions of trypsin residues with chebulagic acid. (c) The interactions of trypsin residues with catechin. (d) The interactions of trypsin residues with piperine.

The analysis of the trypsin-azadirachtin docked complex (Figure 3(a)) shows that the residues Ser195 (1.93 Å) and Gly216 (2.63 and 2.20 Å) show the H-bonding interactions (Figure 3(a) and Table 3) with the azadirachtin. The residues His57, Tyr94, Ser190, Cys191, Gln192, Gly193, Asp194, Asp217, Gly219, Trp217, and Val213 make van der Waals interactions, and residues Arg96 and Leu99 make alkyl and pi-alkyl type of interactions as shown in Figure 3(a).

The analysis of trypsin-chebulagic acid docked complex (Figure 3(b)) shows that the residues Try94 (Å), Ser190 (1.83 Å), Ser195 (0.00 Å), Gly219 (1.99 and 20.1 Å), and Cys220 (2.65 Å) shown in Figure 3(b) and Table 3. Besides, the residues phe41, Cys58, Lys60, Arg96, and Tyr172 make van der Waals interactions and residues Val227, His57, Val213, Gly226, Gly216, and Leu99 form carbon-hydrogen bonds, while the residues Cys191 and Trp 215 make amide-pi-stacked interactions shown in Figure 3(b). Next, docked complex Tyrpin-catechin shows that the residues Ser190 (2.11 and 2.93 Å), Asp189 (2.65 Å), and Phe41 (2.35 Å) make hydrogen bonding interactions shown in Figure 3(c) and Table 3. The residues Cys42, Asp194, Val213, Val227, and Try228 make van der Waals interactions while the Ser195 makes Pi-lone pair and Gly216 makes unfavorable donor-donor interactions (Figure 3(c)); the residues Cys191 and Trp215 form amide-Pi stacked, and Cys220 makes the Pi-alkyl type of interactions as shown in Figure 3(c). Finally, the trypsin-piperin docked complex shows that the residues Ser190 (2.54 Å) and Gln192 (2.25 Å) make hydrogen bonding interactions (Figure 3(d) and Table 3). While the residues such as Asp194, Ser195, Trp215, Ser214, Val213, Gly226, Val227, Tyr228, Ala221, Asp17, Tyr172, and Cys191 make van der Waals interactions and the Cys220 forms Pi-sulfur, Cys42, Cys58, and His57 makes an Alkyl and Pi-alkyl types of interactions (Figure 3(d)).

The docking analysis shows that the compound azadirachtin, chebulagic acid, catechin, and piperine prefer the same binding site of the trypsin receptor protein. These compounds form conventional and nonconventional hydrogen bonding interactions with the trypsin receptor, as shown in Figures 3(a)–3(d) and Table 3.

## 4. Discussion

Oral diseases continue to be a global health issue, and caries and periodontal diseases seem to be the most commonly encountered dental problems. There is a global need for preventive and therapeutic measures and products for oral diseases that are safe, economical, and efficacious compared to the currently used chemotherapeutics with various adverse effects. Oral diseases such as gingivitis and periodontitis represent the specific inflammatory response to microbial residents of the subgingival biofilm. Periodontal disease (PD) is an inflammatory condition affecting the periodontium, including the gingiva, cementum, periodontal ligament, and alveolar bone, representing tooth-supporting structures. It is estimated that 10–15% of the world's population is affected by PD due to a complex interaction between many factors such as genetic and epigenetic factors, age, sex, smoking, and systemic health. A recent study revealed a significant positive correlation between cytokines and a highly negative correlation between vitamin D levels and salivary cytokine levels. There is evidence that the host's inflammatory response facilitates tissue destruction and variability of host responses that cause variability in clinical manifestations of periodontal diseases, ultimately resulting in teeth loss [28, 29]. Therefore, managing such conditions possessing potent anti-inflammatory properties, many chemical plaque control agents have been used to treat the same, including cetylpyridinium chloride, chlorhexidine, and amine fluorides. These have reported that side effects such as staining of the teeth, alteration of taste, and ethanol-containing mouthwashes are associated with oral cancer [30].

The primary goal of ethnopharmacology is to develop efficient nutraceuticals from active plant compounds with the least or no adverse effects. These active compounds from the plant extracts can be subsequently transformed into potential medications that can prevent and treat most dental inflammatory conditions and infections [31].

*Azadirachta indica*, commonly known as *neem*, has been used to treat various diseases since time immemorial. This is owing to its medicinal properties, including antibacterial, anticariogenic, antihelminthic, antidiabetic, antioxidant, astringent, antiviral, cytotoxic, and anti-inflammatory activities [32, 33]. It possesses various active compounds responsible for its beneficial properties. *Neem* bark is an active ingredient in severalkinds of toothpaste and toothpowders. *Neem* bark is known to possess antibacterial properties in dentistry for treating gingival problems and maintaining oral health [34]. There is increasing evidence of *Terminalia chebula* in managing dental caries and periodontal disease [35, 36]. *Piper nigrum*, commonly known as black pepper/kali miri, has been suggested to prevent dental caries and treat halitosis, gingivitis, and periodontitis with deep periodontal pockets [37]. Camellia sinensis, commonly known as green tea, is known for its antimicrobial and antioxidant properties, which could effectively manage oral afflictions such as dental caries, periodontal diseases, and halitosis. However, the studies in dental applications are still evolving with limited authentication. Owing to the active compounds in all the mentioned plant extracts, this study explored their anti-inflammatory potential by protease inhibition, a mechanism on which there is minimal literature. The study also explored the binding mode and interaction of trypsin protein with the active ingredients in the plant extracts, which could be responsible for the anti-inflammatory activities.

According to Leelaprakash and Mohan-Dass [38], neutrophils are a rich source of serine proteinase and are localized at lysosomes. It was previously reported that leukocyte proteinase plays an essential role in developing tissue damage during inflammatory reactions, and a significant level of protection was provided by protease inhibitors [38], like flavonoids. Prostaglandins and nitric oxide biosynthesis are involved in inflammation, and isoforms of inducible nitric oxide synthase (iNOS) and cyclooxygenase (COX-2) are responsible for the production of a significant amount of these mediators.

Various recent studies have shown that many flavonoids contributed significantly to many plants' antioxidant and anti-inflammatory activities. Therefore, bioactive compounds in these leaves may contribute to their anti-inflammatory activity [39]. It has been demonstrated that flavonoids can inhibit both enzymes and other mediators of the inflammatory process, such as reactive C protein or adhesion molecules. However, data related to antiprotease activity is minimal.

Similarly, the current investigation results correlate that, during the in vitro study, the trypsin molecule is considered a mediator molecule for inflammatory reaction. The plant extracts used in the current study are anti-inflammatory agents providing an optimal level of protection acting as protease inhibitors. During the proteinase inhibition assay, it is believed that the plant's extract binds to the trypsin molecule, denoting that the inflammatory activity is to be subsided.

In this study, we have evaluated the potential anti-inflammatory activity of the traditionally used plants *Azadirachta indica*, *Terminalia chebula*, *Camellia sinensis*, and *Piper nigrum*. It is observed that these plant parts have potential anti-inflammatory activity as it shows significant protease inhibition activity. Further, the docking analysis also shows that the active ingredient has significant binding affinity through hydrogen bonding, van der Waals, and *π*-type of interactions as shown in Figures 2(a) and 2(b) and Table 3.

### 4.1. Limitations

However, the isolation, purification, and identification of active ingredients from the abovementioned plants and further inhibition assay against the targeted protein could help to identify the potential compounds for the treatment of oral disease.

## 5. Conclusion

In this study, the anti-inflammatory potential was depicted clearly in protease inhibition assays for the aqueous extract of medicinal plants *Azadirachta indica*, *Terminalia chebula*, *Camellia sinensis*, and *Piper nigrum*. Furthermore, the molecular docking studies of active ingredients of the plants mentioned above reveal that these compounds prefer a similar binding site and potentially inhibit the protease activity of the trypsin enzyme. All the compounds make hydrogen bonding, van der Waals, and other types of interactions with trypsin which could help to stabilize the trypsin-drug complexes and inhibits its function further. Hence, *in vitro* and computational study shows that these active ingredients potentially inhibit the proteinase activity and could be further used as an anti-inflammatory drug and further used to design and develop anti-inflammatory compounds, which could be targeted to manage dental inflammatory conditions.

## Figures and Tables

**Table 1:** Yield of the aqueous extract of *Azadirachta Indica*, *Terminalia chebula*, *Piper nigrum*, and *Camellia sinensis.*

Plant name	Dried material (gm)	Extract (gm)	Colour	Extract yield (%)
*Azadirachta indica*	16	3.22	Brownish solid	20.12
*Terminalia chebula*	16	2.28	Brownish solid	14.25
*Piper nigrum*	16	3.10	Brownish solid	19.37
*Camellia sinensis*	16	3.96	Brownish solid	24.75

**Table 2:** *In vitro* anti-inflammatory activity of aqueous extracts of selected medicinal plants by protease inhibition method.

Plant name	Conc (*μ*g mL^−1^)	OD	% inhibition	IC50
Blank	—	2.138	—	—

Standard (diclofenac sodium)	100	1.221	42.89	93.00
	200	0.905	57.67	
	400	0.851	60.19	
	800	0.768	64.07	
	1000	0.704	67.07	

*Azadirachta indica* Linn	100	1.211	43.35	96.19
	200	0.938	56.12	
	400	0.819	61.69	
	800	0.774	63.79	
	1000	0.689	67.77	

*Piper nigrum* Linn	100	1.481	30.72	371.20
	200	0.969	54.67	
	400	0.912	57.34	
	800	0.827	61.31	
	1000	0.811	62.06	

Terminalia chebula	100	1.869	12.58	639.48
	200	1.752	18.05	
	400	0.969	54.67	
	800	0.861	59.72	
	1000	0.715	66.55	

*Camellia sinensis* Linn	100	1.121	47.56	188.50
	200	1.010	52.75	
	400	0.990	53.69	
	800	0.877	58.98	
	1000	0.645	69.83	

Values represented in the results are mean ± SD of three replicates; linear regression analysis was used to calculate IC50 value).

**Table 3:** Hydrogen bonding interactions of trypsin with active ingredients of azadirachtin, chebuligenic acid, catechin, and piperine after molecular docking.

Protein-drug complex	Binding energy (kcal/mol)	Atoms involved in bonding	Distance (Å)	Angle (°)	Figure reference
Azadirachtin	-5.93	GLY216:N-LIG1:O	2.63642	116.175	2A
		LIG1:H-SER195:OG	1.93069	113.056	
		LIG1:H-GLY216:O	2.11922	138.919	

Chebuligenic acid	-6.14	LIG1:H-A:GLY219:O	1.99686	100.082	2B
		LIG1:H-A:CYS220:SG	2.79062	154.215	
		LIG1:H-A:GLY219:O	2.01555	133.026	
		LIG1:H-A:SER190:OG	1.83137	115.943	
		LIG1:H-A:CYS220:SG	2.65444	131.571	

Catechin	-7.57	LIG1:H-SER190:OG	2.11049	112.514	2C
		LIG1:H-ASP189:OD1	2.65182	130.387	
		LIG1:H-SER190:O	2.93003	115.032	
		LIG1:H-PHE41:O	2.35007	145.123	

Piperine	-8.36	SER190:HG-LIG1:O	2.54118	100.688	2D
		GLN192:HE21-LIG1:O	2.25269	108.11	

Lig was denoted for the drug.

## Data Availability

All research-related data can be made readily available on reasonable request.
